# Supplementary material for: Developmental Vitamin D Deficiency Produces Behavioral Phenotypes of Relevance to Autism in an Animal Model
Source: Nutrients. 2019 May 27;11(5):1187. doi: 10.3390/nu11051187 (PMC6566814; doi:10.3390/nu11051187)
Supplement: Supplementary file 1 [file nutrients-11-01187-s001.pdf]

Supplementary material

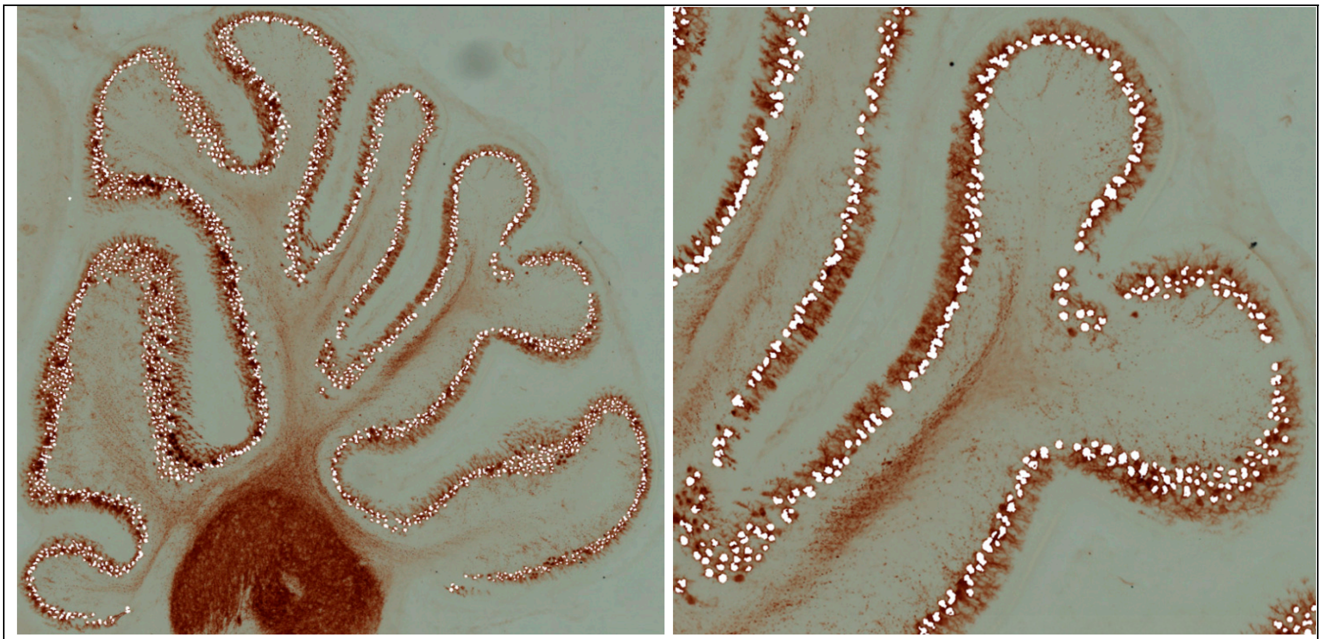

Figure S1. *Cerebral Purkinje cell layers in a P10 rat brain. These figures show the cerebral Purkinje cell layers in dark brown color with light background. Segmentation algorithms were used to distinguish the Purkinje cells from their background. This lead to binary version of data with pixels either classified as cells or non-cells (background).*

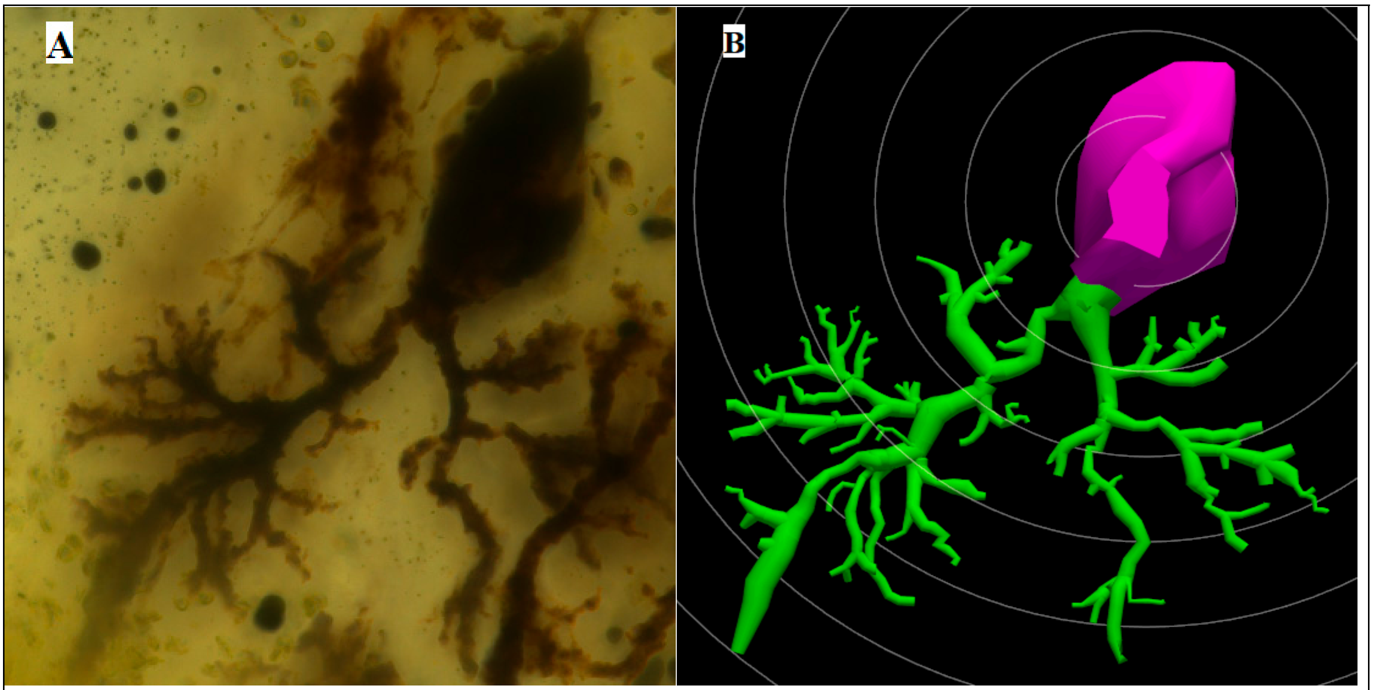

***Figure S2**. Dendritic morphology of a Purkinje cell in a P10 rat brain. Image A was taken from a Golgi-Cox stained section at 63X using Zeiss Axio Imager (Zeiss, Germany). Image B is a 3D reconstruction of serial stack of image A. A semi-automated tracing program Neurolucida® (MBF Bioscience, USA) was used for this purpose.*

Supplementary Table S1. Weight gain in DVD-deficient and control animals.

Body weight (g)				
**Groups**	**Postnatal day 3**	**Postnatal day 7**	**Postnatal day 30**	**Postnatal day 60**
**DVD-deficient males**	8.93 ± 1.33	15.07 ± 1.91	132.5 ± 25.4	301.6 ± 30.6
**Control males**	8.87 ± 1.57	15.45 ± 2.71	129.2 ± 17.9	296.1 ± 27.8
**DVD-deficient females**	8.82 ± 1.12	14.45 ± 1.86	117.2 ± 16.2	194.1 ± 20.3
**Control females**	8.19 ± 1.17	14.04 ± 2.60	121.8 ± 10.8	204.2 ± 11.2

Each data represents mean ± SD

Supplementary Table S2. Appearance of developmental milestones.

**Developmental Milestones**			
**Groups**	Ear unfolding	Fur appearance	Eye opening
**DVD-deficient**	3.59±0.68	5.72±0.72	13.97±1.25
**Control**	3.61± 0.65	5.83±0.77	14.03±1.28

Data represent the postnatal day on which each of above developmental milestone appeared/completed. The ear unfolding and the eye-opening were considered when ear unfolded and eye opened completely, whereas fur appearance was recorded when fur appeared. Each data represents mean ± SD.
